# A research update on the antitumor effects of active components of Chinese medicine ChanSu

**DOI:** 10.3389/fonc.2022.1014637

**Published:** 2022-09-27

**Authors:** Jinhao Jia, Jie Li, Qiusheng Zheng, Defang Li

**Affiliations:** Collaborative Innovation Platform for Modernization and Industrialization of Regional Characteristic Traditional Chinese Medicine, School of Integrated Traditional Chinese and Western Medicine, Binzhou Medical University, Yantai, China

**Keywords:** ChanSu, telocinobufagin, bufotalin, bufalin, cinobufotalin, cinobufagin, antitumor

## Abstract

Clinical data show that the incidence and mortality rates of cancer are rising continuously, and cancer has become an ongoing public health challenge worldwide. Excitingly, the extensive clinical application of traditional Chinese medicine may suggest a new direction to combat cancer, and the therapeutic effects of active ingredients from Chinese herbal medicine on cancer are now being widely studied in the medical community. As a traditional anticancer Chinese medicine, ChanSu has been clinically applied since the 1980s and has achieved excellent antitumor efficacy. Meanwhile, the ChanSu active components (e.g., telocinobufagin, bufotalin, bufalin, cinobufotalin, and cinobufagin) exert great antitumor activity in many cancers, such as breast cancer, colorectal cancer, hepatocellular carcinoma and esophageal squamous cell carcinoma. Many pharmaceutical scientists have investigated the anticancer mechanisms of ChanSu or the ChanSu active components and obtained certain research progress. This article reviews the research progress and antitumor mechanisms of ChanSu active components and proposes that multiple active components of ChanSu may be potential anticancer drugs.

## Introduction

Cancer is a complex disease that affects the life quality and life expectancy of patients globally (1) and is one of the leading causes of death worldwide, with more than 10 million people dying from cancer each year. The hallmarks of cancer include persistent proliferative signaling, evading growth suppressors, resisting cell death, achieving replicative immortality, inducing angiogenesis, and activating invasion and metastasis (2). Fundamentally, cancer results from uncontrolled growth of cells. Radiation therapy and chemotherapy are among the main treatments for cancer at present (3). However, the efficacy of these cancer treatments is not remarkable, and they severely affect the life quality of cancer patients due to pronounced side effects (3). Traditional Chinese medicine may provide new ways to treat cancer and has gradually become an indispensable force in the fight against cancer (4). An increasing number of traditional Chinese medicines are applied against cancer; examples include barbed skullcap herb (*Scutellaria barbata* D. Don.), *Solanum nigrum* L., and Pseudobulb of *Cremastra appendiculata* (5–7). The toxic and side effects of Chinese medicines are relatively small, the efficacy is mild, and the continuity of medication can be maintained, which offers advantages in suppressing tumor angiogenesis as well as preventing and controlling tumor metastasis (8).

ChanSu (also known as toad venom or Venenum Bufonis) is a precious traditional Chinese medicine derived from the dried secretions of the Asiatic toad (*Bufo gargarizans* Cantor) or black-spectacled toad (*Bufo melanostictus* Schneider) of the Bufonidae family (9). The medical records of ChanSu firstly appeared in *Yao Xing Lun* of the Song Dynasty written by Quan Zhen (10) and be mentioned in following dynasties which described the processing method of ChanSu and its applications (11, 12). ChanSu is efficacious in detoxifying, reducing swelling, relieving pain, invigorating the spleen, and inducing resuscitation, and has been used mostly to treat furuncles, carbuncles, sore throat, faint, and infantile malnutrition (13). Historically, ChanSu has been used as a cardiotonic, diuretic, and hemostatic agent (14). With the development of extraction and detection technology, the components of ChanSu have been gradually identified including bufadienolides, indole alkaloids, sterols, epinephrine, proteins, amino acids, polysaccharide, etc. (15). Among these components of ChanSu, bufadienolides and indole alkaloids (bufotenines) were considered as two of the most important bioactive substances (16). Previous studies demonstrated the cyto-toxic activities of bufadienolides and verified in various cancers, including leukemia (Bufalin) (17), hepatocellular carcinoma (Bufothionine) (18), lung carcinoma (cinobufacini) (19), prostatic adenocarcinoma (20), and colon cancer (bufalin and cinobufagin) (21). In additional, bufotenines such as bufobutanoic acid and bufopyramide were demonstrated could inhibit mice P388 lymphocytic leukemia cell (22). Bufothionine, the most focused and widely studied indole alkaloids of ChanSu which have been verified possess notable anti-tumor effect on various cancer cells, including liver cancer (23), gastric cancer (24). Since the pharmaceutical active components of ChanSu and the pathogenic factors of cancers are overly complicated (25), despite an increasing number of medical scientists participating in the research of ChanSu active components, the action mechanisms of a portion of active components remain to be further studied.

This article reviews the latest research results on the modulation of tumor cells by active components of ChanSu *in vitro* and *in vivo*. Moreover, we explore and discuss the regulatory mechanisms of ChanSu active components for distinct cancer types, different signaling pathways, and various molecular targets.

## Cancer and ChanSu active components

### Cancer

Cancers have a genetic basis but are not necessarily hereditary diseases. When normal cells are influenced by carcinogens, genetic changes in the cells can result in the loss of normal regulatory functions for growth, thereby leading to abnormal cell proliferation in the human body. Therefore, drug-induced cancer cell apoptosis (also known as programmed cell death) and inhibition of cancer cell proliferation, migration, and metastasis are the most important means of treating tumors. Dysregulation of apoptosis has been linked to many human chronic disorders, including cancer (26), and inducing apoptosis in tumor cells has been validated as an effective way to treat cancer (27). Most antineoplastic drugs can achieve anticancer effects through modulating apoptosis (28), and the common ones include cisplatin (29), 5-fluorouracil (30), and paclitaxel (31).

However, these chemotherapeutic agents can easily cause drug resistance of tumor cells. With the increased drug dose and prolonged duration of medication, the drug resistance of tumor cells becomes stronger and human body tolerance decreases, with severe side effects emerging, such as hair loss, reduced immune function, nausea, and vomiting. Although combined therapy regimens comprising multiple drugs are utilized in clinical practice, side effects and drug resistance remain major obstacles in cancer therapy (32). Emerging evidence demonstrate that the combined therapy of Chinese herbal medicine and chemotherapeutic drugs may minimize the side effects of chemotherapeutic drugs and maintain a certain continuity in medication, which may have important implications in the suppression of tumor metastasis, enhancement of patients’ physical fitness, and improvement of patients’ quality of life. Especially, ChanSu displays potent antineoplastic activity and has been extensively investigated as a new broad-spectrum anticancer drug.

### ChanSu active components

The extraction and identification studies of ChanSu can be traced back to 1963, after thin layer and paper chromatography tracing, three sterols and 19 bufadienolides were isolated and identified from the skin of Bufo japonicus formosus (33). In the next 60 years, 23 indole alkaloids (34), 142 bufadienolides (15) were extracted and identified from the skin or dried powdered of toad venom. Indole alkaloids of ChanSu, characterized as high hydrophilicity, which are almost derived from serotonin (34). Bufobutanoic acid and bufopyramide were earliest reported indole alkaloids of ChanSu in 1999 (35). In one study, methanol extracts of ChanSu were separated by preparative HPLC with the conditions of H_2_O-Acetonitrile (98:2) solution containing 0.05% TFA and H_2_O-Acetonitrile (85:15-83:17), respectively, and identified by the ^1^H and ^13^C NMR spectral data (35). After that, other 21 indoles alkaloids such as serotonin, N′-methyl-serotonin, bufotenine, bufotenidine, dehydrobufotenine, bufothionine were extracted and identified by various isolation and identification methods including HPLC-ESI-Q-TOF-MS/MS, Phenyl-hexyl column with an ultraviolet detector, preparing thin-layer chromatography and HPLC, and atmospheric pressure chemical ionization tandem mass spectrometry (APCI-MS/MS) (12, 36–39). Unfortunately, scientific researchers seem to prefer extraction to in-depth study on the efficacy of these indoles’ alkaloids. Only a few studies focused on the bio-activities of bufothionine and bufotenine which demonstrated that bufothionine have potential activities in induced cell cycle arrest (G2/M phase arrest), inhibited tumor growth in liver cancer cells (40) and in H22 bearing mice (18). In terms of bufotenine, previous studies verified its powerful anti-inflammatory and psychotropic properties resulted from inhibited nuclear factor-kappa B (NF-κB) signaling pathway and its high affinity for the 5-hydroxytryptamine receptor, respectively (41, 42).

**Figure 1:**
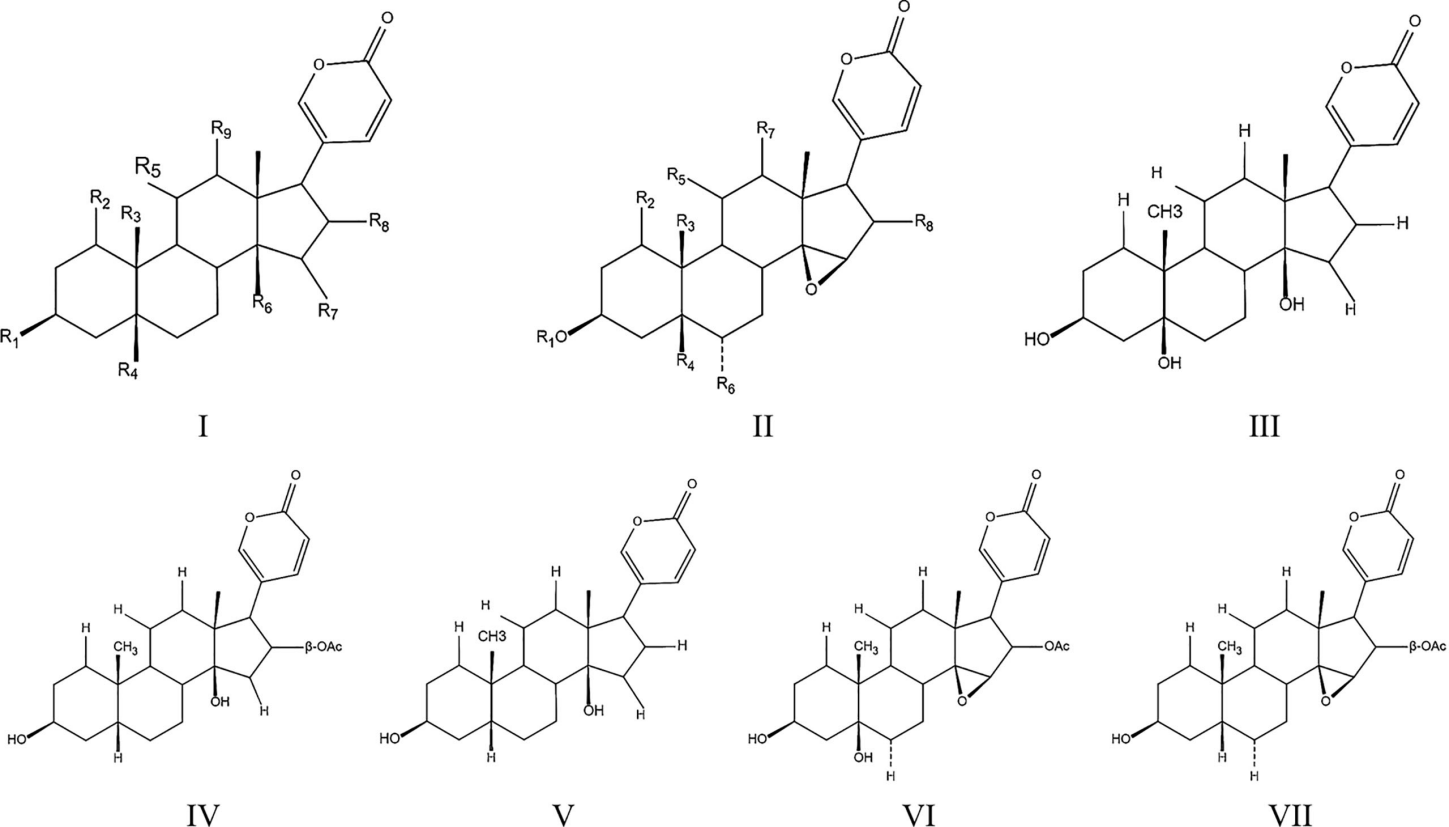
Toadienohydroxylate lactone parent nucleus (I, II), Telocinobufagin (III), Bufotalin (IV), Bufalin (V), Cinobufotalin (VI), Cinobufagin (VII).

Except for indoles alkaloids, bufadienolides are a class of C-24 steroids, which are characterized by a diunsaturated six-membered lactone ring at C-17 (α-β) and considered as the most important components of ChanSu (43, 44) **(** **Figure 1**). In 1965, 19 bufadienolides including bufotalin, bufalin, bufotalinin, telocinobufagin, and cinobufotalin were isolated by thin layer and paper chromatography and identified from the skin of the Japanese toad bufo (33). Next, another 16 bufadienolides (such as resibufogenin, hellebrigenin, desacetylbufotalin, gamabufotalin, and cinobufagin) were isolated from chloroform extracts of ChanSu and subjected to silica gel chromatography (45, 46). Since that, reversed-phase preparative high-performance liquid chromatography (HPLC), TBE-300 high-speed counter-current chromatography, and ultra-high performance liquid chromatography (UHPLC) methods were used to separate bufadienolides from ethanol, methanol, and water extracts of ChanSu and identified by NMR or quadrupole time-of-flight mass spectrometry (Q-TOF/MS) technique (47–49). Meanwhile, anti-tumor activities of ChanSu bufadienolides were testified in various cancer cells. For example, bufalin and cinobufagin significantly induced cell apoptosis, cell cycle arrest, inhibit cell migration and invasion in liver cancer cells (50–52). The similar anti-tumor effects of ChanSu bufadienolides were observed in lung cancer (bufalin and gamabufotalin) (53, 54), osteosarcoma (cinobufagin, bufalin and bufotalin) (55, 56), and colorectal cancer (cinobufagin, resibufogenin, and doxorubicin) (57, 58). Among them, five bufadienolides including telocinobufagin, bufotalin, bufalin, cinobufotalin, and cinobufagin are the most focused and studied ChanSu bufadienolides and their anti-tumor activities have been verified in various cancers (**Table 1**).

**Table 1:** The antitumor activities and mechanisms of five bufadienolides.

Compounds	Subjects (cells/animals)	Concentration	Safe dose for animals	Research Mechanisms	Main mechanisms	Tumor Types	References
Telocinobufagin	4T1 cells, breast cancer cells, HCT116 cells, SW480 cells, and BALB/c mice splenocytes	0.1~10 μM or 1, 5, 25 and 125 mg/L	10 or 20 µg/mouse	PI3K/Akt/ERK/Snail signaling pathway PI3K/Akt pathway MMP-2, MMP-9 synthetic signaling pathway Bax pathway activation Th1 cytokine regulatory pathway	Apoptosis, necrosis, inhibition of proliferation and migration, activation of immune cells	Breast cancer, Colorectal cancer, Potential treatment	(59, 60)
Bufotalin	Hep 3B cells, Hep G2 cells, R-HepG2 cells, U2OS cells, SaOs-2 cells, MG-63 cells, HeLa cells, A375 cells, ESCC cell lines, and BGC-823 cells	0.01~10 μM	0.5 mg/kg	caspases pathway Akt-mediated signal pathway endoplasmic reticulum stress Bid- and STAT1-dependent pathways The mitochondrial apoptotic pathway p53 signaling pathway caspase-3 apoptosis pathway	Apoptosis, cell necrosis, inhibition of proliferation and migration, cell cycle arrest, inhibition of DNA repair, ER stress	Hepatocellular carcinoma, Malignant osteoblastoma, Cervical cancer, Cutaneous malignant melanoma, ESCC, and Gastric cancer	(61–67)
Bufalin	T24 cells, SK-N-BE cells, SH-SY5Y cells, U251 cells, U87MG cells, LN-229 cells, HepG2 cells, BxPc3 cells, and Sw1990 cells	1~300 nM	5 mg/kg	caspase pathway AKT pathway EPK pathway electron transport chain pathway endoplasmic reticulum stress Fas- and mitochondria-mediated pathways TBK1 activation pathway cellular gene transcriptional	Apoptosis, cell necrosis, inhibition of proliferation and migration, cell cycle arrest, enhancement of cell sensitivity, inhibition of metastasis, oxidative stress, ER stress	Bladder cancer, drug-resistant bladder cancer, Neuroblastoma, Glioblastoma, Hepatocellular carcinoma, and Pancreatic cancer	(68–80)
Cinobufotalin	A549 cells, H460 cells, HTB-58 cells, HONE1-EBV cells, 5–8F cells, HepG2 cells, LM3 cells, SMMC7721 cells, KYN-2 cells, Huh-7 cells, SNU-739 cells, and Male SD rats	0.025~10 μM or 20, 200 and 2000 ng/mL	4 mg/kg	mPTP opening pathway EMT signals Pathway MYH9 expression Pathway targeting lipogenesis Induces cells to produce ceramide mitochondrial pathway Regulation of P-glycoprotein	Apoptosis, cell necrosis, inhibition of proliferation and migration, reduction of ATP synthesis, enhancement of cell sensitivity, inhibition of metastasis and invasion	Lung cancer, Nasopharyngeal Carcinoma, and Hepatocellular carcinoma	(81–86)
Cinobufagin	HepG2 cells, MG-63 cells, 143B cells, U2OS cells, SaOS-2 cells, HCT116, RKO cells, SW480 cells, HK-1 cells, and SGC-7901 cells	0.001~1000 µM	1.0 mg/kg	Fas- and mitochondria-mediated pathways Notch signaling pathway IL-6-OPN-STAT3 pathway GSK-3/NF-κB pathway STAT3 pathway Akt/mTORC1/HIF-1α pathway mitochondrial pathway	Apoptosis, cell necrosis, inhibition of proliferation and migration, cell cycle arrest, inhibition of angiogenesis, inhibition of metastasis and invasion, inhibition of autophagy	Hepatocellular carcinoma, Osteosarcoma, Colorectal cancer, Nasopharyngeal Carcinoma, and Gastric cancer	(55, 69, 87–92)

## The anti-tumor mechanisms of five ChanSu bufadienolides

### Telocinobufagin

Telocinobufagin (TBG) is an active component isolated from the traditional Chinese medicine ChanSu and possesses the pharmacological properties of ChanSu, such as immunomodulation (93) and inhibition of Na+/K+-ATPase activities (60). Previous pharmacokinetic studies of TBG in rats demonstrated that after orally administered 120 mg/kg ChanSu extracts, the C_max_ of TBG in plasma was 0.69 ± 0.26 μg/ml which imply its absorption is extremely low but rapid due to the T_max_ was 35 ± 18 min. Meanwhile, 135 ± 43 min T_1/2_ demonstrated that TBG has moderate elimination rate in rats (94). In addition, TBG exhibits important antineoplastic activities, such as inhibiting tumor cell proliferation, constraining cell differentiation, arresting cell cycle, suppressing tumor angiogenesis, and hindering tumor cell metastasis. *In vitro*, telocinobufagin showed powerful cyto-toxic activities in various tumor cells (IC50 in LLC-PK1, A549, and H157 cells were 0.20 µM, 27.882 ± 17.291, and 23.606 ± 7.381 ng/mL, respectively (95). Numerous studies have focused on the antitumor effects of TBG on breast cancer. It has been shown that TBG (0.05 and 0.5 μg/ml) significantly inhibits migration and invasion of breast cancer cells *via* modulate PI3K/AKT/ERK/Snail pathway (59). Evaluation of anti-metastatic effect in a highly metastatic 4T1 mouse model has revealed that the tumor volume in mice after TBG treatment significantly decreased and the mass evidently decreased, without obvious changes in mice body weight, and H&E staining confirmed the lack of metastasis of breast cancer cells in the lungs (59). Further research has demonstrated that TBG represses epithelial–mesenchymal transition (EMT) *via* the Akt/ERK/Snail signaling pathway (59), thereby concluding that the Akt/ERK/Snail signaling pathway is a promising anticancer target of TBG. Additionally, it has been shown that the PI3K/Akt signal transduction pathway is closely related to tumorigenesis and development of breast cancer, and the expression levels of p-PI3K and p-Akt are increased in breast cancer cells under hypoxia, while the expression levels decrease after TBG intervention, suggesting that TBG may exert its function through the PI3K/Akt pathway and suppress the proliferation and invasion of breast cancer cells (59). According to another experiment, TBG can block breast cancer cell mobility through effectively impeding MMP-2 and MMP-9 synthesis, thereby achieving the inhibition of breast cancer cell proliferation and migration (96). Furthermore, some studies have reported that TBG is proficient in suppressing colon cancer cell viability and inducing apoptosis and is capable of significantly raising the necrosis and apoptosis rates of colorectal cancer (CRC) cells. Previous studies have also indicated that the suppression of CRC cells by TBG is related to the p53-mediated Bax pathway (97).

**Figure 2:**
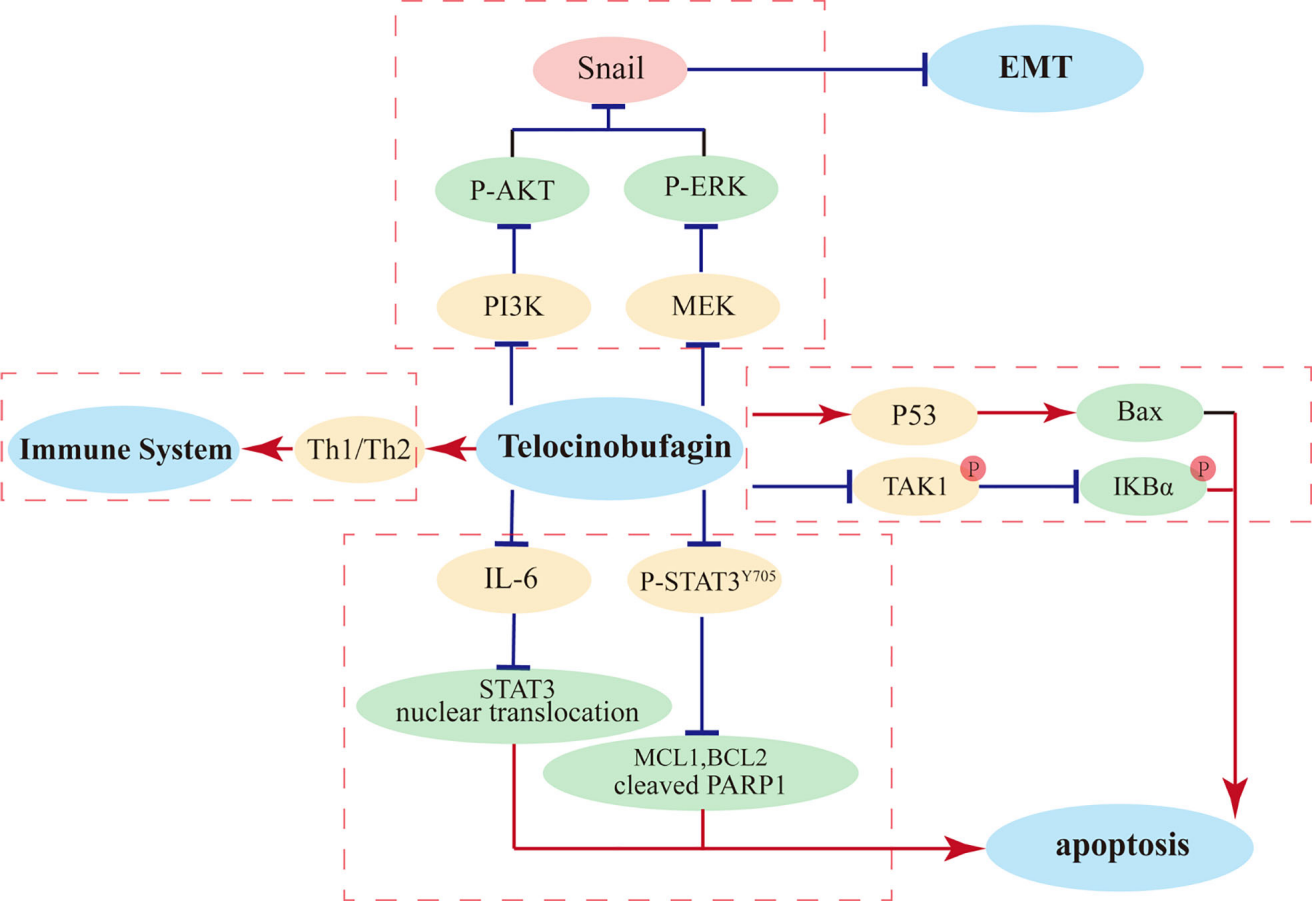
The primary mechanisms for TBG induced anti-tumor effects.

Moreover, TBG can modulate multiple immune cells that play key roles in immune responses. TBG induces the expression of Th1 cytokines and inhibit the secretion of Th2 cytokines, thereby enhancing the killing capacities of natural killer cells and macrophages, which become the first line of defense to protect the host from tumor and viral infection. Thus, TBG can exert anticancer activities through regulating immune cells and can be a potential therapeutic agent to treat cancer (93). The primary anti-tumor mechanisms of TBG in cancer cells were showed in **Figure 2**.

### Bufotalin

Bufotalin is an extract from the traditional Chinese medicine ChanSu prepared from the dried secretion of the auricular and skin glands of Bufo gargarizans Cantor. In Kunming mice, single intravenous injection bufotalin showed rapid distributive and eliminated characters due to the T_1/2_ and MRT of bufotalin in plasma were 28.6 min and 14.7 min, respectively (98). The anti-tumor effects of bufalin have been testified in various cancer cells such as HepG2 Cells (IC_50 =_ 130 ± 10 nM (48 h)), Eca-109 cells (IC_50 =_ 0.8 µM (72 h)) (95). In details, bufotalin is able to induce apoptosis of human hepatocellular carcinoma (HCC) Hep3B cells (99), with experimental data suggesting that the inhibition rate of bufotalin for Hep3B cells could reach 80% (61). Another study showed that multiple active components of ChanSu had evident inhibitory effects on human HCC cell line (HepG2) and its doxorubicin-induced multidrug resistant liver cancer cells (R-HepG2); bufotalin had the best performance among these components and displayed extremely potent anti-hepatoma activity (64). Cell cycle analysis revealed that bufotalin induced apoptosis through the inhibition of the Akt-mediated signal pathway and induced cell cycle arrest at G2/M phase in HepG2 cells with upregulated p53 gene expression, thereby decreasing cellular viability and effectively suppressing the proliferation and migration of HepG2 cells.

It has been shown that bufotalin-induced apoptosis in osteoblastoma cells is associated with endoplasmic reticulum (ER) stress activation (65). Mild ER stress is usually considered a pro-survival and adaptive response, while prolonged or severe ER stress can promote apoptosis (62). After bufotalin treatment, MG63 cells are significantly less proliferative. Further experiments have shown that bufotalin promotes apoptosis *via* the activation of caspase-12, and the use of caspase inhibitors significantly represses the anticancer activity of bufotalin. Bufotalin sensitizes TNF-related apoptosis-inducing ligand (TRAIL)- and tumor necrosis factor-α (TNF-α)-induced apoptosis of HeLa cells. Through sensitizing death receptor–induced apoptosis *via* Bid- and STAT1-dependent pathways, bufotalin enhances chemotherapeutic drugs–induced apoptosis and downregulates antiapoptotic gene expression (63).

A study showed that bufotalin can simultaneously induce cell cycle arrest and apoptosis in A375 cells. Further experiments have revealed that bufotalin induces melanoma cell cycle arrest at G2/M phase *via* upregulation of ATM and Chk2 and downregulation of Cdc25C. Additionally, bufotalin may induce apoptosis *via* the mitochondrial apoptosis pathway and inhibition of AKT phosphorylation, which provides convincing evidence for bufotalin as a potential therapeutic drug for treating malignant melanoma of the skin (67). Another study has indicated that bufotalin induces p53-mediated apoptosis in esophageal squamous cell carcinoma (ESCC) cells. Bufotalin can effectively inhibit the viability of ESCC cells, enhance caspase protein activity, upregulate the expression of DNA damage-associated proteins, inhibit DNA repair, and markedly inhibit the expression of Ki-67, a biomarker of proliferation, suggesting that bufotalin exhibits therapeutic potential for ESCC through modulating the p53 signaling pathway (66). In addition, bufotalin has a proapoptotic effect on human gastric cancer BGC-823 cells (100).

**Figure 3:**
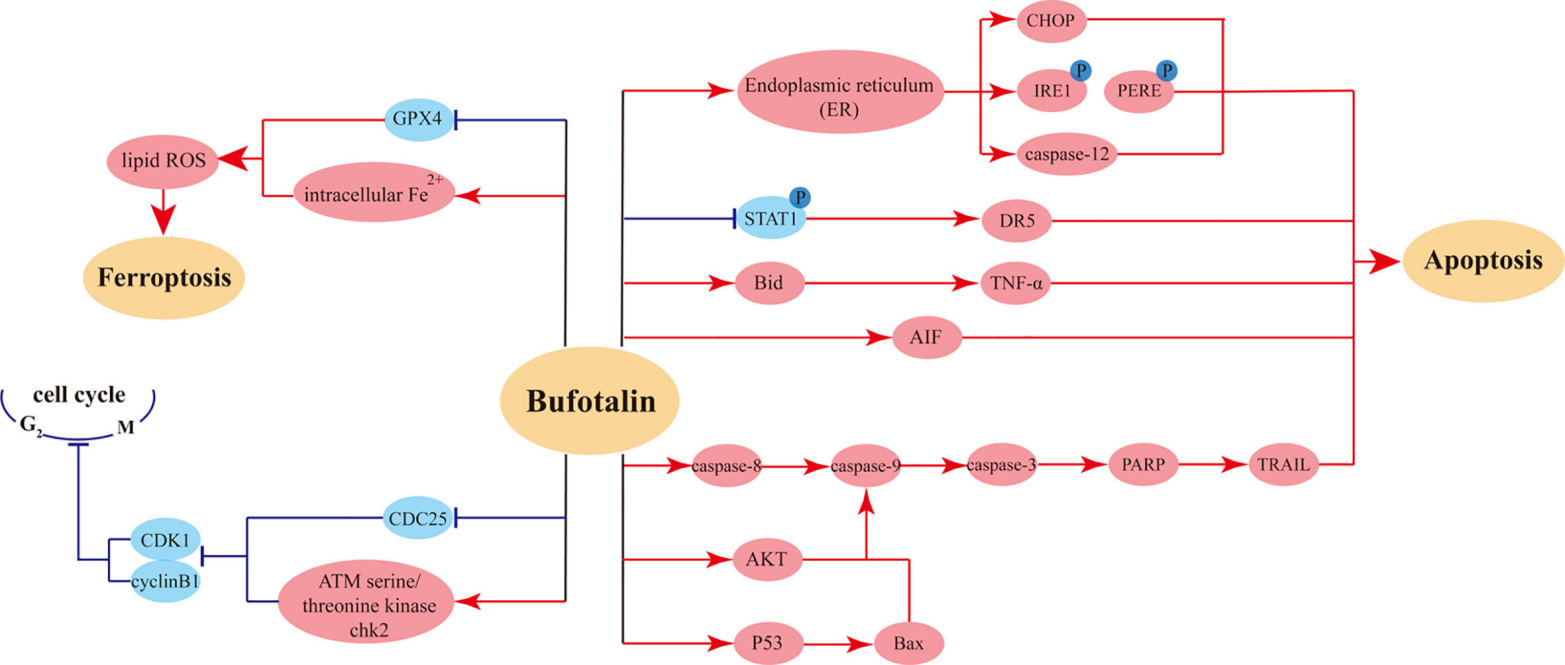
The primary mechanisms for bufotalin induced anti-tumor effects.

Moreover, examination of the pharmacokinetics and tissue distribution following single-bolus injection and constant-rate infusion of bufotalin in mice showed that, except for the lungs and brain, bufotalin concentrations were low in most tissues. Constant-rate infusion resulted in higher bufotalin concentrations in the lungs and brain compared with single-bolus injection within the same time. These results suggest that bufotalin might be a promising antitumor candidate for lung cancer (98). The bufotalin induced mainly anti-tumor effects on cancer cells were showed in **Figure 3**.

### Bufalin

Bufalin is a cardiotonic steroid and a principal component of the traditional Chinese medicine ChanSu extracted from the skins and parotid venom glands of Bufo gargarizans Cantor. After intravenous administration of ChanSu extracts in SD rats (0.8 mg/kg), rabbits (0.35 mg/kg), and beagle dogs (0.18 mg/kg), the T_1/2_ of bufalin were 24.32 ± 3.78, 21-31, and 14-28 min, respectively (101–103). In additional, oral administration of 10 mg/kg bufalin in SD rats, the pharmacokinetic parameters in plasma demonstrated that the T_max_ and t_1/2_ were 22.50 ± 8.02 and 375.76 ± 243.85 min, respectively, which imply that bufalin have rapid absorptive and slowly eliminated properties (104). Numerous studies have reported that bufalin possesses very potent antitumor activities, and it is deemed one of the most valuable anticancer drugs. However, the mechanisms of bufalin’s antineoplastic actions are not fully understood. The mechanisms of bufalin effects on human bladder cancer are being particularly studied. It has been shown that bufalin can induce apoptotic cell death in human bladder cancer cells through the activation of both the intrinsic and extrinsic pathways. Bufalin induces cell cycle arrest at the G2/M phase, inhibits the growth of human bladder cancer cells, and suppresses cell proliferation, invasiveness, and metastasis. Additionally, it can induce apoptosis by activating the mitochondria-mediated intrinsic caspase pathway and the death receptor–mediated extrinsic pathway (70). Another study has reported that bufalin induces G0/G1 phase arrest in human bladder cancer T24 cells by reducing the levels of cyclin D, cyclin E, CDK2, and CDK4, and triggers apoptosis through the mitochondrial signaling pathway (71). Experimental data have also revealed that bufalin treatment results in repressed Akt activity and an increase in the proapoptotic Bax caused by dissociation from antiapoptotic Bcl-2, thereby leading to mitochondrial dysfunction, cytochrome c release, activation of caspase cascades, and consequently apoptosis in bladder cancer cells (71).

The experimental results have also shown that the antitumor effect of bufalin is related to the phosphorylation of the extracellular signal-regulated protein kinase (ERK) pathway, and the inhibitory effect of bufalin on T24 cell invasion may be partially fulfilled *via* the ERK pathway activation (72). Another study has demonstrated that bufalin can sensitize human bladder carcinoma cells to TRAIL-mediated apoptosis (76). TRAIL can induce programmed cell death (68, 74). TRAIL exerts minimal impact on normal cells but can selectively induce apoptosis of numerous transformed or malignant cells (73). However, certain malignant tumor cells are resistant to TRAIL. Combined usage of bufalin and TRAIL can markedly inhibit the viability of human bladder carcinoma cells, sensitizing the TRAIL-resistant bladder carcinoma cells to TRAIL-mediated apoptosis. Overall, bufalin may be a new anticancer drug in the future with features of low toxicity and rare side effects, which may provide a better therapeutic effect for patients with drug-resistant bladder cancer.

With regard to the treatment of neuroblastoma, researchers have constructed a chemically modified bufalin probe CS-P1 that retains the antitumor activity of bufalin in neuroblastoma, which is able to inhibit the proliferation and migration of neuroblastoma cells. Further investigation has confirmed that the antitumor effect of bufalin in neuroblastoma *in vitro* is achieved by targeting the electron transport chain (ETC). The accumulation of reactive oxygen species (ROS) induced by the disruption of the ETC decreases the mitochondrial membrane potential, increases the permeability of mitochondrial membrane, and causes the build-up of cytochrome c in cytoplasm, thereby inducing apoptosis (80). Furthermore, in glioblastoma, bufalin can augment the radiosensitivity of glioblastoma by repressing mitochondrial function and DNA damage repair (77) and can also improve the sensitivity to temozolamide (TMZ) (79), which provides the theoretical basis for the combined therapy of bufalin and radiation.

**Figure 4:**
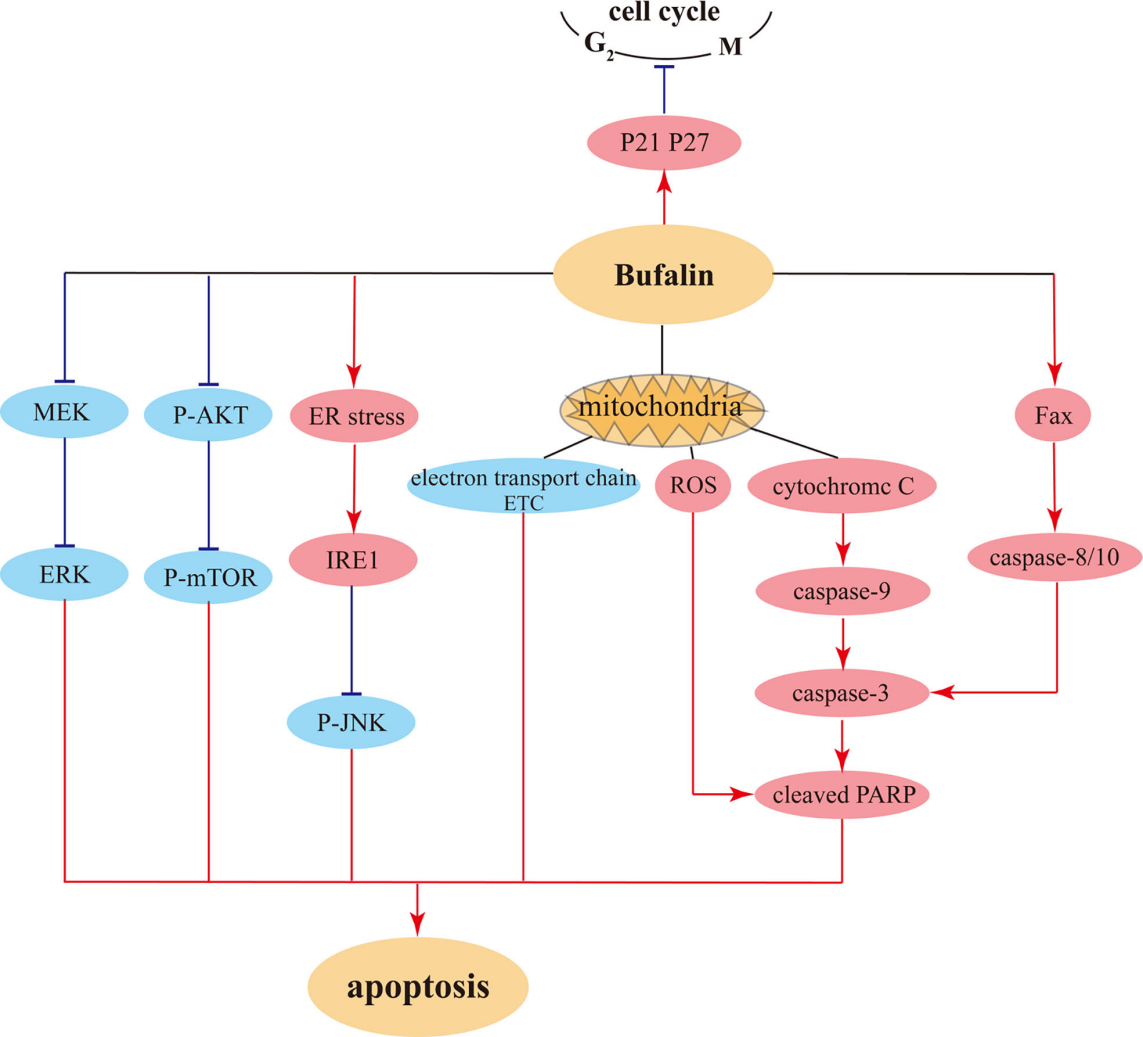
The possible pathways for bufalin induced cell cycle arrest and pro-apoptosis in tumor cells.

There are also research findings showing that bufalin induces apoptosis of human HCC cells through the Fas- and mitochondria-mediated pathways, and a caspase-10-dependent, Fas-mediated pathway might play a more crucial role (69). There are also related reports on the suppression of side effects from liver cancer chemotherapeutic drugs (78). Furthermore, bufalin has a certain inhibitory influence on pancreatic cancer, which is the most difficult tumor to target. Namely, bufalin exhibits strong inhibitory effect on pancreatic cancer cell proliferation both *in vitro* and *in vivo*, and induces cell cycle arrest at S phase *via* upregulating p27 level, consequently promoting apoptosis (75). The illustration of bufalin-triggered pathways that involved in tumor cell cycles and apoptosis were showed in **Figure 4**.

### Cinobufotalin

Cinobufotalin (CB) is a bufadienolide discovered in toad venom. Previous *PK* studies used ChanSu extracts were performed in SD rats demonstrated that compared with intravenous injection (0.8 mg/kg), CB concentrations in oral administrated (120 mg.kg) rats plasma showed comfortable elimination rate (T_1/2_ of intravenous injection was 22.58 ± 5.58 min vs. 119 ± 26 min of oral administration). Meanwhile, oral administration exhibited rapid but low absorptive rate (C_ma_x and T_max_ were 0.68 ± 0.33 μg/ml, and 17 ± 15 min, respectively) (94). Several studies have confirmed that CB possesses anticancer activities (81). The research on CB has never stopped, among which nasopharyngeal and liver cancers were mostly investigated. It has been revealed for the first time that EBV-miR-BART22 directly targets MAP2K4 and stimulates Myh9 expression, thereby inducing the ubiquitin degradation of GSK3β protein and consequently promoting tumor stemness, metastasis, and cisplatin chemoresistance *via* the activation of β-catenin-induced stemness and EMT signals (84). Further investigation has revealed that CB can powerfully reverse EBV-miR-BART22-induced cisplatin resistance *via* upregulating MAP2K4 to antagonize Myh9/GSK3β/β-catenin and its downstream tumor stemness and EMT signals in nasopharyngeal carcinoma. It has been proposed that the elevated miR-BART22 and decreased MAP2K4 expression may be important markers to predict poor prognosis in patients with nasopharyngeal carcinoma (84). It has also been found that CB can effectively induce FOXO1-stimulated cisplatin sensitivity by antagonizing FOXO1’s binding partner Myh9 (83). High Myh9 expression promotes the invasion and metastasis of tumor cells (105, 106), and CB suppresses tumor invasiveness *via* inhibiting Myh9 expression. Both *in vitro* and *in vivo* experiments have suggested that CB can improve the sensitivity of FOXO1-overexpressing nasopharyngeal carcinoma cells to cisplatin and promote their apoptosis, indicating that CB is a potential drug to fight against nasopharyngeal carcinoma (83).

With regard to the research on CB in the treatment of liver cancer, as a novel SREBP1 inhibitor, CB suppresses HCC proliferation by targeting lipogenesis, which significantly reduces the lipid levels in HepG2 cells and inhibits carcinoma growth, consequently exerting antitumor influence (86). In addition, another study has shown that CB induces HCC cells to produce ceramide, and ceramide production mediates CB-induced growth inhibition and apoptosis in HCC cells. It has been proposed that SphK1 suppression, ceramide production, and Akt/mTORC1 inactivation may be responsible for the inhibitory impact of CB on HCC cells (82). Scholars have also proposed that CB can induce apoptosis of liver cancer cells *via* the mitochondrial pathway.

**Figure 5:**
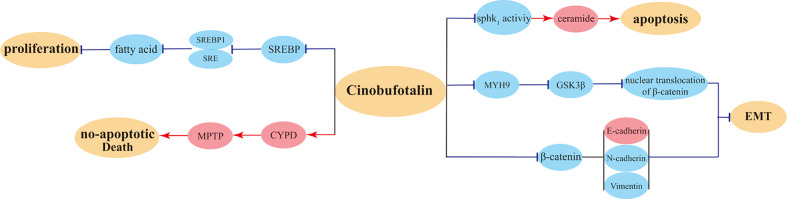
Cinobufotalin (CB) induced mainly anti-tumor pathways.

A recent study has acquired data on CB-treated breast cancer cells from the Gene Expression Omnibus (GEO) database and has analyzed the profile of differentially expressed genes; the study suggested that CB may exert anticancer activities in MCF-7 cells in similar ways as miconazole. CB can suppress the proliferation of SKOV3 cancer cells through decreasing ARF6 protein expression, thereby accomplishing antitumor effect (107). Another animal experiment validated that as a substrate of P-glycoprotein, the distribution of CB in animal liver tissues can be modulated by P-glycoprotein. This suggests that the combined application with P-glycoprotein inhibitor can increase the accumulation of CB in plasma and liver in the treatment of liver cancer, thereby augmenting its antitumor efficacy (85). Overall, CB induced mainly anti-tumor pathways were showed in **Figure 5**.

### Cinobufagin

Cinobufagin is one of the principle active components in the traditional Chinese medicine ChanSu and an effective traditional Chinese medicine monomer extracted from *Bufo gargarizans* Cantor and *Bufo melanostictus* Schneider (108). Similar as CB, the *PK* parameters of cinobufagin showed its absorptive and eliminated rates were less than 30 min (the T_max_ of oral administration 120 mg/kg ChanSu extracts was 20 ± 12 min) and great than 2 hours (T_1/2_ was 138 ± 30 min), respectively, although with weak absorptive amount (C_max_ was 0.77 ± 0.12 μg/ml) (94). Numerous studies testified the anti-tumor cell properties of cinobufagin, such as U2OS cells (IC_50 =_ 100 nM, 48 h), SMMC-7721 Cells (IC_50 =_ 92 ng/mL, 48 h), EC9706 cells (IC_50 =_ 3.2 µM, 72hrs), Hec2 cells (IC_50 =_ 2.4 µM, 72hrs), and MCF7 Cells (IC_50 =_ 0.44 ± 0.12 µM, 48 h) (95). A study reported that cinobufagin was able to decrease the viability of OS cells and induce their apoptosis both *in vitro* and *in vivo*. Under the influence of cinobufagin, Notch-1 gene was downregulated, indicating that cinobufagin may induce apoptosis of OS cells through the inactivation of the Notch signaling pathway (55). Another study showed that cinobufagin may inhibit the characteristics of OS cancer cells by suppressing the IL-6-OPN-STAT3 pathway and may also inhibit the metastasis and invasiveness of OS cells. Additionally, when OS cells were repressed, the normal human osteoblast hFOB1.19 cells were not obviously impacted (90). There was also research showing that cinobufagin manifested potent antitumor activities by inducing G2/M phase arrest and apoptosis in OS cells. Further mechanistic research has suggested that cinobufagin-induced apoptosis is partially achieved through the suppression of the GSK-3/NF-κB pathway (87). To sum up, cinobufagin is likely to be a promising drug candidate for OS treatment.

Moreover, regarding the research on the action mechanisms of cinobufagin in the inhibition of CRC, experimental data have indicated that cinobufagin evidently suppresses CRC cell viability *via* the STAT3 pathway inhibition, promotes apoptosis, and represses proliferation and migration of CRC cells. Besides, cinobufagin showed therapeutic effects in a CRC xenograft model without damaging major organs (92). Cinobufagin can also impede the growth of CRC cells by suppressing angiogenesis (109). Further experimental results have indicated that cinobufagin can inhibit angiogenesis in CRC through downregulating the Akt/mTORC1/HIF-1α pathway and triggering MOMP-mediate apoptosis, consequently reaching antitumor outcome (88). Thus, cinobufagin is a very promising biotherapeutic drug to treat CRC.

**Figure 6:**
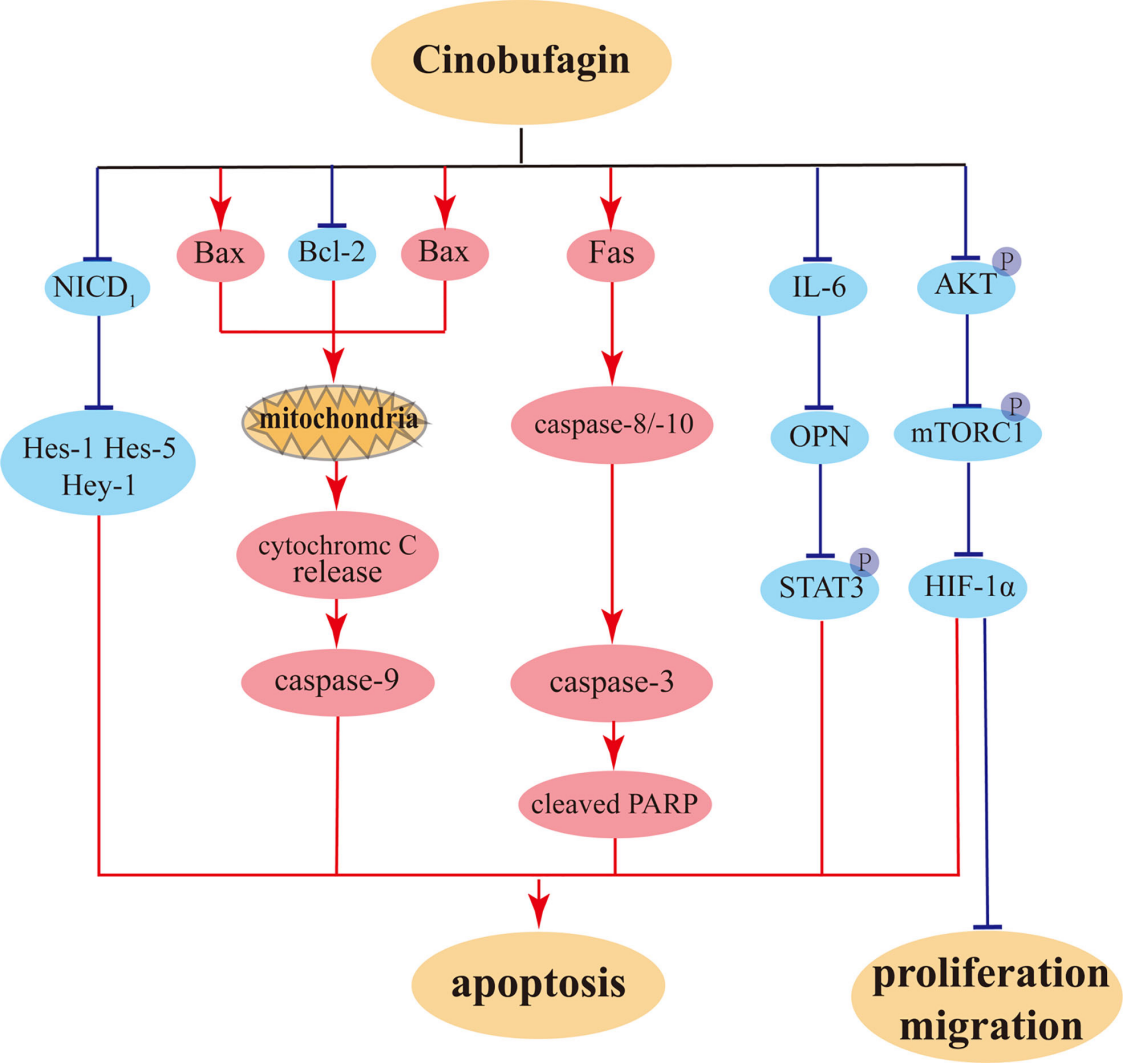
The potential anti-proliferation, anti-migration, and pro-apoptosis pathways of cinobufagin on tumor cells.

Cinobufagin can also promote apoptosis in nasopharyngeal carcinoma cells. By downregulating the levels of CDK2 and cyclin E, cinobufagin induces cell cycle arrest at the S phase in HK-1 cells and increases ROS levels, leading to DNA damage (110). In addition, cinobufagin promotes apoptosis in nasopharyngeal carcinoma HK-1 cells through downregulating the protein levels of Bcl-2 and the apoptotic protein PARP1 (91). It has also been shown that cinobufagin-induced apoptosis in gastric cancer is enhanced *via* inhibition of autophagy (111, 112). At the same time, cinobufagin can induce ROS production, further augment the effect of autophagy inhibition, increase proapoptotic gene expression, and disrupt mitochondrial membrane potential, thereby inducing apoptosis in gastric cancer cells (89). The illustration of primary anti-tumor effects of cinobufagin on cancer cells were showed in **Figure 6**.

## Clinical applications of cinobufacini combination therapy

It has been reported that the anti-tumor effects of various active ingredients of Chansu were significantly better than those of other anti-tumor drugs, such as the herbal extract paclitaxel and the chemotherapeutic drug cisplatin (32, 113). The combination of Chansu active ingredients and other chemotherapeutic drugs is significantly more effective than the single application of two drugs, and also increases the sensitivity of drug-resistant cells to chemotherapeutic drugs (114). As a traditional medicine in China, the Chinese medicine extracts of ChanSu have been made into cinobufacini injection for clinical application. Cinobufacini is an aqueous injection solution processed from the shaded dried skin of the traditional Chinese medicine *Bufo gargarizans* Cantor and has been approved by the China Food and Drug Administration as a chemotherapeutic drug to treat liver and prostate cancers in China (115). Previous studies investigated the chemical constituents in cinobufacini injection by UPLC-ESI-QTOF/MSE method and the results showed that 76 components were identified including 12 alkaloids, 13 peptides, 36 bufadienolides, and 15 other components (such as organic acids, amides, and sterols). In terms of bufadienolides, telocinobufagin, bufotalin, bufalin, cinobufotalin, and cinobufagin were identified (116). Similarly, these five components were also identified in cinobufacini treated rats plasma (117).

Cinobufacini injection showed satisfied properties in detoxification, swelling reduction, pain relief, and illnesses including middle and advanced stage tumors as well as chronic hepatitis B. Clinical study data have shown that combination application of cinobufacini with radiotherapy and chemotherapy evidently elevates efficacy compared with chemotherapy (gemcitabine-oxaliplatin) alone in patients with locally advanced or metastatic gallbladder carcinoma, and mitigates the toxic or side effects of radiation and chemotherapy (118). Another study indicated a possible effective component in cinobufacini that could exert anti-tumor effect in HCC patients (40). In the treatment of a case of advanced lung cancer with malignant pericardial effusion, after intrapericardial cinobufacini instillation, the patient’s pain was significantly relieved and life quality improved with hardly any adverse reactions, which provided a new approach for the treatment of patients with advanced cancer who cannot tolerate chemotherapeutic drugs (119). In the treatment of primary liver cancer after transarterial chemoembolization (TACE), intra-arterial infusion of cinobufacini combined with sorafenib competently improved short-term antitumor efficacy, reduced the levels of inflammatory cytokines and tumor markers, and was well tolerated with minimal adverse reactions (120). Clinical adjuvant therapy with cinobufacini in elderly patients with colorectal cancer enhanced short-term efficacy of chemotherapy, largely reduced the toxic or side effects of radiotherapy and chemotherapy, and improved the body’s immune function, exhibiting high clinical application values (121).

## Future research perspectives

The incidence and mortality of cancer are increasing each year, and treatments with chemotherapy or radiation alone generate little desirable outcomes, which is why finding new ways to fight cancer is imperative. In this situation, traditional Chinese medicine, which has been passed on for thousands of years, is gradually being increasingly recognized by most physicians and patients. Years of clinical applications confirm the definite antitumor effect of ChanSu, and cinobufacini in combination with other chemotherapeutic drugs shows very good clinical efficacy, which has great implications for the scientific and rational utilization of cinobufacini and full employment of the unique antitumor effect of traditional Chinese medicine.

In addition, several studies showed that Chansu has adverse effects such as cardiotoxicity (122), hepatotoxicity (123), and central system toxicity (124). Chansu also can rapidly alter intracellular calcium stores Na^+^/K^+^ concentrations in cardiac myocytes, causing them to stop beating within seconds (125). However, the cardiac toxicity of ChanSu could be effectively alleviated through intravenous infusion, which provides an efficient way to take advantage of the multiple extracts of ChanSu in clinical application (126). At present, clinical research on ChanSu is primarily conducted in China, with rare related clinical studies conducted abroad. Future developments should aim at enhancing antitumor activities, identifying antitumor mechanisms, and reducing toxic side effects. A greater number of clinical studies should be carried out under the premise of safe medication in order to ensure the clinical efficacy and absolute safety of ChanSu and its effective active components.

## Conclusion

This article reviews the antitumor effects of the five active components of the traditional Chinese medicine ChanSu and their molecular mechanisms. Previous research has revealed that the inhibition of tumor cells by these active components is achieved through multiple pathways, and the primary anticancer mechanisms include inducing cancer cell apoptosis, inducing autophagy, arresting cell cycle, suppressing metastasis, repressing growth, and reversing drug resistance. A substantial amount of evidence indicates that these active components may become potential drugs for clinical treatment of cancers in the future. Before clinical application, further experimental studies are needed to elucidate their exact molecular mechanisms. Overall, ChanSu and its active compounds can antagonize cancer cells through a variety of mechanisms, inhibit cancer cell proliferation, migration, and differentiation, and promote cancer cell apoptosis (17).

The clinical application of many chemotherapeutic agents can cause numerous serious adverse reactions, leading to unsatisfactory clinical outcomes. Therefore, anticancer agents derived from natural resources may become the most promising agents for cancer treatment. As a representative of antitumor drugs in traditional Chinese medicine, ChanSu is broadly utilized in clinical practice. However, the composition of compounds extracted from ChanSu is complex with a lack of detailed clinical trial processes. The toxicity of ChanSu has not been supported thorough experimental investigation, and there are still many gaps to be filled regarding the anticancer aspects.

Gaining a better understanding of tumorigenesis and the pharmacological effects of the natural medicine ChanSu, as well as its active components, will be helpful for the large-scale clinical studies on these drugs and their targets in the future. This article reviews the research on the therapeutic effects of ChanSu active components in the treatment of cancer, in order to provide theoretical support and medication guidance in their clinical treatment.

## Author contributions

Conceptualization: JL and DL; data curation: JJ; writing – original draft: JL, JJ, and DL; writing – review and editing: QZ and DL. All authors contributed to the article and approved the submitted version.

## Funding

This research was funded by the National Natural Science Foundation of China (82073313 to DL, 31870338 to QZ), Taishan Scholars Construction Engineering of Shandong Province (to DL), the Yantai High-End Talent Introduction Plan “Double Hundred” (to DL).

## Conflict of interest

The authors declare that the research was conducted in the absence of any commercial or financial relationships that could be construed as a potential conflict of interest.

## Publisher’s note

All claims expressed in this article are solely those of the authors and do not necessarily represent those of their affiliated organizations, or those of the publisher, the editors and the reviewers. Any product that may be evaluated in this article, or claim that may be made by its manufacturer, is not guaranteed or endorsed by the publisher.
